# Sobrevida em 10 Anos de Pacientes com Insuficiência Cardíaca com FEVE 40-59%: Uma Classificação Fenotípica Viável?

**DOI:** 10.36660/abc.20210772

**Published:** 2023-01-31

**Authors:** Luiz Claudio Danzmann, Luiz Carlos Bodanese, Aline Petracco Petzold, Anna Paula Tscheika, Ellen Hettwer Magedanz, Lucas Celia Petersen, Evgeny Belyavskiy, Fernanda Lourega Chieza

**Affiliations:** 1 Universidade Luterana do Brasil Canoas RS Brasil Universidade Luterana do Brasil, Canoas, RS – Brasil; 2 Hospital São Lucas da PUCRS Porto Alegre RS Brasil Hospital São Lucas da PUCRS, Porto Alegre, RS – Brasil; 3 Santa Casa de Misericórdia de Porto Alegre Porto Alegre RS Brasil Santa Casa de Misericórdia de Porto Alegre, Porto Alegre, RS – Brasil; 4 Pontifícia Universidade Católica do Rio Grande do Sul Porto Alegre RS Brasil Pontifícia Universidade Católica do Rio Grande do Sul, Porto Alegre, RS – Brasil; 5 Charité Universitätsmedizin Berlin Berlin Alemanha Charité Universitätsmedizin Berlin, Berlin – Alemanha

**Keywords:** Insuficiência Cardíaca, Mortalidade, Sobrevida, Volume Sistólico

## Abstract

Os limites da fração de ejeção do ventrículo esquerdo (FEVE) para a insuficiência cardíaca (IC) com FEVE levemente reduzida (ICFElr) têm sido questionados, já que evidências demonstram que alguns medicamentos utilizados para IC com FEVE <40% (ICFEr) demonstram eficácia também em populações com FEVE < 60%. Objetivo do estudo foi comparar a sobrevida total e cardiovascular de pacientes com IC com FEVE 40-59% com paciente com ICFEr e IC com FEVE ≥ 60%. Foram incluídos pacientes com IC descompensada que preencheram os critérios diagnósticos de Framingham na admissão hospitalar entre 2009 e 2011. Os pacientes foram divididos em ICFEr, IC com FEVE 40-59% e IC com FEVE ≥ 60%. O método de Kaplan-Meier foi usado para detectar a sobrevida geral e cardiovascular em 10 anos. A significância estatística foi estabelecida em p <0,05. Foram incluídos 400 pacientes, com idade média de 69 ± 14 anos. A sobrevida cardiovascular nos pacientes com IC e FEVE 40-59% não foi diferente em comparação aos pacientes com ICFEr [Hazard Ratio (HR) ajustado 0,86 – Intervalo de Confiança (IC) 95% 0,61-1,22; Ptrend = NS], mas foi estatisticamente diferente em comparação aos com FEVE ≥ 60% (HR ajustado = 0,64 - IC95% 0,44-0,94; Ptrend = 0,023). Não houve diferença na taxa de sobrevida de 10 anos entre diferentes grupos de FEVE. O grupo de pacientes com IC e FEVE ≥ 60% teve maior sobrevida cardiovascular que os outros grupos.

## Introdução

A insuficiência cardíaca (IC) é uma síndrome, cujo manejo baseia-se na classificação da fração de ejeção do ventrículo esquerdo (FEVE). As diretrizes recentes^1 , 2^ propõem o seguinte modelo de fenótipos: IC com FEVE reduzida (<40%) (ICFEr), IC com FEVE levemente reduzida (40-49%) (ICFElr) e IC com FEVE preservada (≥ 50%) (ICFEp). No entanto, o limite superior para o fenótipo ICFEIr ainda é discutido no contexto de quais são os critérios adequados de normalidade para a FEVE.^3^

Análises de ensaios clínicos sugerem os pacientes com ICFElr têm beneficio semelhante com os tratamentos comprovadamente eficazes para ICFEr.^4 , 5^A análise pré-especificada do estudo PARAGON-HF^6^ descreve redução de desfechos clínicos em pacientes com FEVE > 45% e ≤ 57% com sacubitril-valsartan, reforçando a hipótese de que um limite superior mais alto para ICFElr poderia ser adequado para a previsão de resultados.^4^

O objetivo do presente estudo é comparar a sobrevida total e cardiovascular de pacientes com IC com FEVE 40-59% com a da população com ICFEr e com IC com FEVE ≥ 60% em um período de 10 anos de seguimento.

## Métodos

Estudo de seguimento de uma coorte, no qual foram incluídos pacientes com > 18 anos com diagnóstico de IC por critérios de Framingham, confirmados por ecocardiograma, no período entre janeiro de 2009 e dezembro de 2011 e acompanhados por 10 anos. A população foi dividida em três grupos de FEVE: ICFEr, IC com FEVE 40-59% e IC com FEVE ≥ 60%.

Para rastrear a sobrevida do paciente, efetivou-se busca nos prontuários médicos ou por contato telefônico. Para aqueles com inconsistência dos dados, foi realizada busca no Registro Civil do Estado.

Os desfechos avaliados foram: sobrevida total e a sobrevida livre de desfechos cardiovasculares (infarto agudo do miocárdio, rehospitalização por IC, acidente vascular cerebral e arritmias) durante o tempo de acompanhamento.

Análise estatística: para comparar as características quantitativas basais, utilizou-se o teste ANOVA, e o teste de Kaplan-Meier foi realizado para avaliar a sobrevida. O teste de log-rank foi usado para determinar as diferenças nas distribuições de sobrevivência, seguido por regressão de Cox univariada e multivariada, ajustada para idade, hipertensão, diabetes mellitus, doença arterial coronariana, índice de massa corporal, doença pulmonar obstrutiva crônica e doença renal crônica. Foi obtido um valor de *Hazard Ratio* (HR) com seus respectivos intervalos de confiança (IC) de 95%. P <0,05 foi considerado para indicar significância estatística.

## Resultados

Da população inicial de 423 pacientes provenientes da região metropolitana de Porto Alegre, 400 foram incluídos (133 com FEVE<40, 145 com FEVE 40-59% e 122 com FEVE≥60%). 60,1% apresentavam classe funcional III/IV da New York Heart Association.

**Figura 1:**
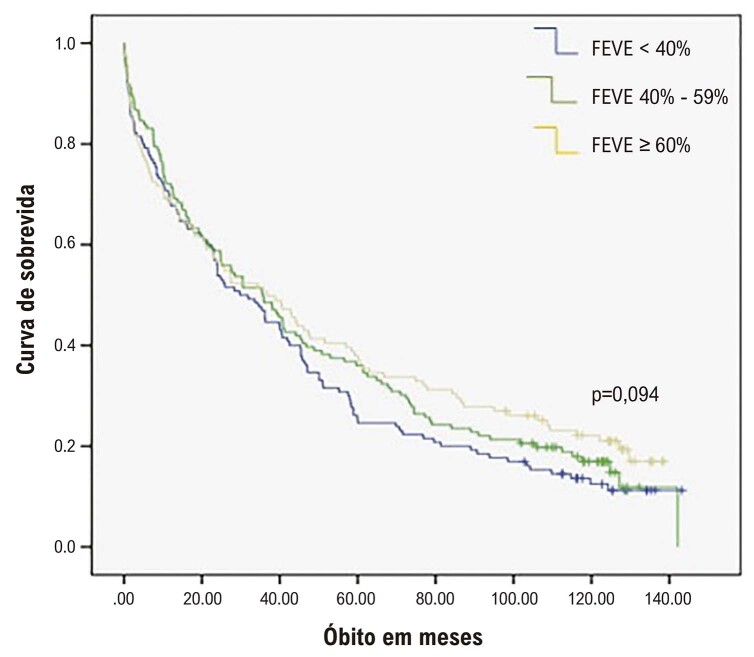
– Curva de Kaplan-Meier para morte por todas as causas. FEVE: fração de ejeção do ventrículo esquerdo.

Dos pacientes acompanhados, 324 (81%) pacientes morreram. A taxa de sobrevida foi de 32,8% em 5 anos. Nenhuma associação estatisticamente significativa foi demonstrada entre os grupos de IC estratificados por FEVE < 40%, FEVE 40-59% e FEVE ≥ 60% e a mortalidade total ( Figura 1 ).

Na análise de Kaplan-Meier, observa-se que os pacientes com ICFEr tiveram uma sobrevida mediana de 4,5 anos, seguidos por IC com FEVE 40-59% (5,7 anos) e por IC com FEVE ≥ 60% (8,8 anos).

**Figura 2:**
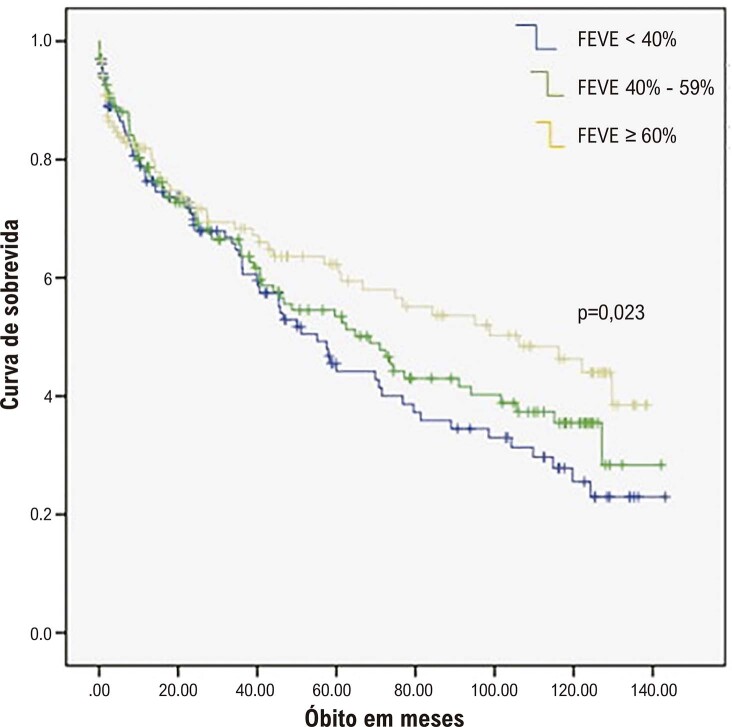
– Curva de Kaplan-Meier para morte por causas cardiovasculares. FEVE: fração de ejeção do ventrículo esquerdo.

A análise univariada e multivariada ajustada para idade, hipertensão, DM, DAC, IMC, DPOC e DRC. Em relação à sobrevida cardiovascular, o grupo ICFE 40-59% somente foi estatisticamente diferente em comparação com ICFE ≥ 60% (HR ajustado = 0,64 - IC 95% 0,44-0,94, Ptrend = 0,023) ( Tabela 1 , Figura 1 e Figura 2 ).

**Tabela 1:** – Mortalidade geral e cardiovascular

	Mortalidade Geral		Mortalidade Cardiovascular	
Grupos	HR não ajustado	HR ajustado*	HR não ajustado	HR ajustado*
FEVE 40-59%	0,89(0,69-0,15)	0,89 (0,68-1,17)	0,86 (0,62-1,20)	0,86 (0,61-1,22)
FEVE ≥ 60%	0,80 (0,61-1,07)	0,78 (0,59-1,04)	0,68 (0,48-0,98)	0,64 (0,44-0,94)
p de tendência	0,125	0,094	0,039	0,023

* Estimativa obtida por regressão de Cox adaptada aos seguintes atributos: idade, hipertensão, diabetes mellitus, doença arterial coronariana, índice de massa corporal, doença pulmonar obstrutiva crônica e doença renal crônica. FEVE: fração de ejeção do ventrículo esquerdo; HR: hazard ratio.

## Discussão

Nossos resultados não demonstraram diferença significativa na sobrevida entre o fenótipo ICFE 40-59% e os outros grupos de FEVE. Esse resultado reproduz os de outros estudos com populações de pacientes internados por IC, os quais não demostraram a FEVE como marcador de sobrevida total.^7 , 8^ A sobrevida cardiovascular foi significativamente maior no grupo FEVE ≥ 60% que nos outros grupos de FEVE, o que corrobora resultados de registros com populações semelhantes.^8^

A maioria das características dos pacientes com FEVE de 40-59% teve distribuição intermediária em relação aos outros grupos, mas houve maior prevalência de DAC nos ICFEr e com FEVE 40-59% em comparação com FEVE ≥ 60% (43,8% x 53,5% x 64,4%, respectivamente; Ptrend = 0,004). Este fato já foi associado à menor sobrevida cardiovascular em populações com IC e disfunção sistólica.^7^

A baixa taxa de prescrição das medicações modificadoras de prognóstico para IC retratam o sério problema da aplicação das diretrizes na prática clínica.^7^ Entretanto, as diferenças observadas entre os grupos em nosso estudo são semelhantes às observadas em um recente registro europeu.^8^

Por fim, a plausibilidade da ideia de redimensionar o ponto de corte da FEVE da ICFElr para cima também pode ser reforçada pelos dados do recente estudo EMPEROR-Preserved^9^ que demonstrou a eficácia da empagliflozina versus placebo para redução de desfechos de morte cardiovascular e/ou hospitalização em pacientes com FEVE>40%, mas com maior tamanho de efeito no subgrupo com FEVE até 60%, ainda que o P de interação entre os grupos tenha sido não significativo. Esse resultado configura um alinhamento epidemiológico com os nossos dados e dos outros estudos já mencionados.^4 - 8^

## Conclusão

Os resultados do seguimento de 10 anos da nossa coorte de pacientes com IC demonstrou que a sobrevida geral dos pacientes com FEVE 40-59% não diferiu dos dois outros grupos e, em relação à sobrevida cardiovascular, foi significativamente menor que a de pacientes com FEVE≥60%. Esses dados sugerem plausibilidade epidemiológica para a rediscussão sobre limites de FEVE para o fenótipo ICFElr.
