# Supplementary material for: Sobrevida em 10 Anos de Pacientes com Insuficiência Cardíaca com FEVE 40-59%: Uma Classificação Fenotípica Viável?
Source: Arq Bras Cardiol. 2023 Jan 31;120(1):e20210772. [Article in Portuguese] doi: 10.36660/abc.20210772 (PMC10389085; doi:10.36660/abc.20210772)
Supplement: Translation

# Translation: Ten-Year Survival of Heart Failure Patients with Left Ventricular Ejection Fraction of 40-59%: A Potential Phenotypic Classification?

**Authors:** Luiz Claudio Danzmann, Luiz Carlos Bodanese, Aline Petracco Petzold, Anna Paula Tscheika, Ellen Hettwer Magedanz, Lucas Celia Petersen, Evgeny Belyavskiy, Fernanda Lourega Chieza

**Affiliations:** 1 Brazil Universidade Luterana do Brasil, Canoas, RS – Brazil; 2 Brazil Hospital São Lucas da PUCRS, Porto Alegre, RS – Brazil; 3 Brazil Santa Casa de Misericórdia de Porto Alegre, Porto Alegre, RS – Brazil; 4 Brazil Pontifícia Universidade Católica do Rio Grande do Sul, Porto Alegre, RS – Brazil; 5 Germany Charité Universitätsmedizin Berlin, Berlin – Germany

**Keywords:** Heart Failure, Mortality, Survival, Stroke Volume

## Abstract

Definitions of left ventricular ejection fraction (LVEF) cut-off values for HF with mildly reduced LVEF (HFmrEF) have been a subject of debate, in the face of evidence that some drugs used in the treatment of HF with LFEV < 40% (HFrEF) are also effective in patients with LVEF < 60%. The aim of this study was to compare overall survival and cardiovascular survival in HF patients with LVEF of 40-59% in patients with HFrEF and HF with LVEF ≥ 60%. Patients with decompensated HF who met the Framingham diagnostic criteria at hospital admission between 2009 and 2011 were included. Patients were divided into HFrEF, HF with LVEF 40-59%, and HF with LVEF ≥ 60%. The Kaplan-Meier was used to determine ten-year overall survival and cardiovascular survival. The statistical significance was established at p<0.05. A total of 400 patients were included, with a mean age of 69 ± 14 years. Cardiovascular survival in patients with HF and LVEF of 40-59% was not significantly different than in patients with HFrEF (adjusted Hazard Ratio [HR] 0.86; 95% Confidence Interval [CI] 0.61-1.22, Ptrend = NS), but was statistically different compared with patients with LVEF ≥ 60% (adjusted HR of 0.64; 95% CI 0.44-0.94, Ptrend = 0.023). No difference was found in 10-year survival between the LVEF groups. Patients with HF and LVEF ≥ 60% had significantly higher cardiovascular survival compared with the other groups.

## Introduction

The management of heart failure (HF) patients is based on left ventricular ejection fraction (LVEF) classification. Recent guidelines^1 , 2^suggest the following phenotypes of HF: HF with reduced LVEF (<40%) (HFrEF), HF with mildly reduced LVEF (40-49%) (HFmrEF), HF with preserved LVEF (≥50%) (HFpEF). However, the upper limit for HFmrEF is still a matter of debate regarding the most appropriate criteria of normality for LVEF.^3^

Clinical studies have suggested that patients with HFmrEF have similar benefits from therapies shown to be effective for HFrEF.^4 , 5^Prespecified analysis of the PARAGON-HF^6^study reveals a reduction in clinical outcomes in patients with LVEF > 45% and ≤ 57% with sacubitril/valsartan, reinforcing the hypothesis that a higher upper limit for HFmrEF could be more adequate for result prediction.^4^

The aim of the present study was to compare overall and cardiovascular survival between HF patients with LVEF of 40-59% and HF patients with LVEF ≥ 60% in a 10-year follow-up period.

## Methods

This was a cohort study of adult patients (>18 years old) with diagnosis of HF according to the Framingham criteria, confirmed by echocardiogram between January 2009 and December 2011, who were followed for 10 years. The study population was divided into three groups – HFrEF, HF with LVEF 40-59% and HF with LVEF ≥ 60%.

Patient survival was assessed by review of medical records or by telephone contact. In case of data inconsistency, a search in the civil registration system was made.

The following outcomes were evaluated during the follow-up period: overall survival and survival free of cardiovascular outcomes (acute myocardial infarction, rehospitalization for HF, stroke, and arrhythmias).

### Statistical analysis

Comparisons of quantitative characteristics at baseline were made using the ANOVA test, and the Kaplan-Meier test was used for survival analysis. The log-rank test was used to determine differences in survival distribution, followed by univariate and multivariate Cox regression, adjusted for age, hypertension, diabetes mellitus, coronary artery disease (CAD), body mass index (BMI), chronic obstructive pulmonary disease (COPD) and chronic renal disease (CRD). Hazard ratio (HR) values and respective 95% confidence interval (CI) was determined, and a p<0.05 was defined as statistically significant.

## Results

Of the initial sample of 423 patients from the metropolitan region of Porto Alegre, 400 were included (133 with LVEF<40, 145 with LVEF 40-59% and 122 with LVEF ≥60%); 60.1% had New York Heart Association functional class III/IV. A total of 324 (81%) died. The five-year survival rate was 32.8%. No statistically significant association was found between the LVEF groups and overall mortality ( Figure 1 ).

**Figure 1:** – Kaplan-Meier curve for all-cause mortality. LVEF: left ventricular ejection fraction.

In the Kaplan-Meier analysis, patients with HFrEF had a median survival of 4.5 years, patients with LVEF of 40-59% had a median survival of 5.7 years, and patients with LVEF ≥ 60% had a median survival of 8.8 years.

Univariate and multivariate analysis adjusted for age, hypertension, DM, CAD, BMI, CPOD and CRD was performed. Regarding cardiovascular survival, statistically difference was observed between the LVEF 40-59% and LVEF ≥ 60% groups only (adjusted HR = 0.64; 95% CI 0.44-0.94, Ptrend = 0.023) ( Table 1 , Figure 1 and Figure 2 ).

**Table 1:** Overall and cardiovascular mortality

	Overall mortality		Cardiovascular mortality	
Groups	HR (not adjusted)	HR (adjusted)*	HR (not adjusted)	HR (adjusted)*
LVEF 40-59%	0.89 (0.69-0.15)	0.89 (0.68-1.17)	0.86 (0.62-1.20)	0.86 (0.61-1.22)
LVEF ≥ 60%	0.80 (0.61-1.07)	0.78 (0.59-1.04)	0.68 (0.48-0.98)	0.64 (0.44-0.94)
Tendency p value	0.125	0.094	0.039	0.023

*Cox regression adjusted for age, hypertension, diabetes mellitus, coronary artery disease, body mass index, chornic obstructive pulmonary disease and chronic renal disease. LVEF: left ventricular ejection fraction; HR: hazard ratio.

**Figure 2:** – Kaplan-Meier curve for cardiovascular mortality. LVEF: left ventricular ejection fraction.

## Discussion

Our results did not show significant differences in survival between the groups of patients categorized by LVEF. This result reproduces findings from other studies on populations of patients hospitalized for HF, which also did not describe LVEF as a marker of overall survival.^7 , 8^Cardiovascular survival was significantly higher in the group of patients with LVEF ≥ 60% compared with the other LVEF groups, which corroborates previous studies with similar populations.^8^

Most characteristics of patients with LVEF 40-59% showed intermediate distribution as compared with the other groups. However, the prevalence of CAD in patients with HFrEF and LVEF 40-59% was higher compared with patients with LVEF ≥ 60% (43.8% x 53.5% x 64.4, respectively; Ptrend = 0.004). This fact had already been associated with lower cardiovascular survival in populations with HF and systolic dysfunction.^7^

The low rate of prescription of prognostic-modifiers drugs for HF reflects a serious difficulty in applying guideline recommendations in clinical practice.^7^Nevertheless, the differences detected between the groups in our study are similar to those reported in a recent European registry.^8^

Finally, the plausibility of the idea of raising the cut-off values of LVEF in HFrEF is also supported by results of the recent EMPEROR-Preserved study^9^that showed greater efficacy of empagliflozin over placebo in reducing the outcomes of cardiovascular death and/or hospitalization in patients with LVEF>40%. The effect was greater in the subgroup of patients with LVEF<60%, even though the interaction p between the groups was not statistically significant. This result configures an epidemiological alignment with our findings and results of previously mentioned studies.^4 - 8^

## Conclusion

Results of the 10-year follow-up of our cohort of HF patients demonstrated that, in patients with LVEF 40-59%, the overall survival was not different from that in the other two LVEF groups, and cardiovascular survival was significantly lower than in patients with LVEF ≥60%. These data suggest epidemiological plausibility for redefinition of the LVEF cutoffs for HFmrEF.
